# Basic blood biochemical parameters of wild common ravens (*Corvuscorax*)

**DOI:** 10.3897/BDJ.11.e103271

**Published:** 2023-05-15

**Authors:** Tzvetan Chaprazov, Rusko Petrov, Dobri Yarkov, Yana Andonova, Ivanka Lazarova

**Affiliations:** 1 Trakia University, Stara Zagora, Bulgaria Trakia University Stara Zagora Bulgaria; 2 Green Balkans - Stara Zagora NGO, Stara Zagora, Bulgaria Green Balkans - Stara Zagora NGO Stara Zagora Bulgaria

**Keywords:** wild bird health, ravens, blood biochemistry, rehabilitation centre

## Abstract

Baseline haematological and biochemical blood parameters in healthy wild birds are key to managing wild populations and to saving critically ill individuals. This knowledge is crucial for the care, rehabilitation and the release of birds after treatment in wildlife rescue centres. Plasma levels provide valuable information for the evaluation of the physical condition of animals. The objective of this study was to obtain reference values of some basic biochemical blood parameters of wild common ravens (*Corvuscorax*). Between 2020 and 2023, we took blood samples from the wild population of common ravens in Bulgaria (n = 36). We determined the values of 18 parameters - alanine transaminase (ALT, U/I), albumin (g/l), alkaline phosphatase (ALP, U/I), amylase (U/I), aspartate transaminase (AST, U/I), calcium (mmol/l), chloride (mmol/l), cholesterol (mmol/l), creatine kinase (CK, U/I), creatinine (μmol/l), blood glucose (mmol/l), lactate dehydrogenase (LDH, U/I), magnesium (mmol/l), phosphorus (mmol/l), total bilirubin (μmol/l), total protein (g/l), triglycerides (TG, mmol/l) and uric acid (μmol/l). We made a comparative analysis including the regions in which the groups were sampled and the time of year. Most of the presented results were comparable to published values of other species from the Corvidae family and some were higher (ALP, amylase, AST, CK, total protein and uric acid levels). Most of these could be explained by the capture- and handling stress. This is the first report in official literary sources presenting some basic biochemical blood parameters of healthy wild common ravens in Bulgaria. The results may be of use to scientists, veterinarians and other researchers in rescue and rehabilitation centres and they can provide the basis for further studies with regards to animal welfare and health assessment of the species.

## Introduction

The common raven (*Corvuscorax*) is the largest bird of the Crow family (Corvidae) (del Hoyo et al. 2016). Its body length is between 54 and 78 cm and it has a wingspan from 100 to 130 cm (Simeonov et al. 1991). Its weight can reach up to 1.6 kg (Cornell Lab of Ornithology 2023). The common raven has wide and long wings with well-developed primaries and a relatively long tail (Spasov et al. 2006). The plumage is all-black, including the beak and legs. It is widespread in Europe, Asia, North America and North Africa, but it is not abundant anywhere (Marzluff 2009). It lives mainly in the mountains at a considerable elevation. It is rare in Bulgaria and is included as a protected species in the Red Data Book of the Republic of Bulgaria (Golemanski 2011). The common ravens are unusually intelligent, they can count, they have a good memory and there are known cases of learning words.

Changing habitats negatively affect the population (Restani et al. 2001). Conservation efforts can be aided by having sufficient information on the health status of birds in the wild (Ibañez et al. 2015). Nevertheless, there are no studies considering the range for haematology and blood chemistry parameters of the common raven. There is a lack of such baseline clinical studies for captive common ravens as well. There are only a few publications about some of the other representatives of the Crow family. Clinical evaluation of the serum biochemical profile is important for the diagnosis of various diseases due to its prognostic value regarding the degree of damage to various internal organs like heart, liver, kidneys, digestive system etc. (Campbell 2004, Huhtamo et al. 2007, Ihedioha et al. 2010). On the other hand, ravens’ feeding habits make them potential carriers of human and animal pathogens. Several reports have linked them to the spread of avian influenza virus (Khawaja et al. 2004, Tanimura et al. 2016). They can also be a reservoir and a source of human pathogenic bacteria, such as *Salmonella* and *Campylobacter* and viruses (human influenza virus and orthoreovirus) (Huhtamo et al. 2007, Ryall and Meier 2008). This lack of information may deter veterinarians from performing proper evaluations of the health and welfare status of the species.

The goal of this research was to determine the baseline blood biochemical indexes for wild common ravens in Bulgaria in helping to preserve their populations in nature. This information could be used as a complementary tool for veterinary medicine in wildlife rehabilitation centres.

## Material and methods

The research was carried out on 36 wild ravens in the Republic of Bulgaria in the period 2020-2023. The birds were captured individually with respect to humane measures aimed at stress reduction when working with wild birds. The ravens were caught in adaptation aviaries for griffon vultures (*Gypsfulvus*) and cinereous vultures (*Aegypiusmonachus*), part of projects funded by the EU LIFE Programme with aim of restocking and reintroduction of those species in the country. The project “Recovery of the Populations of Large European Vultures In Bulgaria” LIFE08 NAT/BG/000278 was implemented by Green Balkans - Stara Zagora NGO in partnership with the Fund for Wild Flora and Fauna (FWFF, Bulgaria) and the Bird of Prey Protection Society (BPPS, Bulgaria), during which 210 griffon vultures were released between 2010 and 2015. During 2015-2022, Green Balkans - Stara Zagora NGO together with FWFF, The Vulture Conservation Foundation (VCF, Netherlands), Junta de Extremadura (Spain) and EuroNatur (Germany) conducted project “Bright Future for Black Vulture in Bulgaria” LIFE14 NAT/BG/649, aiming for the recovery of the cinereous vulture in Bulgaria.

All common ravens were examined upon blood collection by a veterinary physician from the Wildlife Rehabilitation and Breeding Centre - Green Balkans, part of Green Balkans - Stara Zagora NGO. The birds were determined to be clinically healthy. Surfaces were disinfected with Desclean solution. We disinfected the area and collected 1.5 ml of whole blood from either the left or right basilic vein (*Vena cutanea ulnaris superficialis*) of all specimens tested. We immediately placed the blood into collection tubes containing lithium heparin. We used 3 ml syringes with 23G needles. We transported the samples to a laboratory, where they were processed within 4 hrs of collection using a BS-120 (Mindray, China) biochemical analyser.

We compared the values of the following 18 biochemical parameters - alanine transaminase (ALT, U/I), albumin (g/l), alkaline phosphatase (ALP, U/I), amylase (U/I), aspartate transaminase (AST, U/I), calcium (mmol/l), chloride (mmol/l), cholesterol (mmol/l), creatine kinase (CK, U/I), creatinine (μmol/l), blood glucose (mmol/l), lactate dehydrogenase (LDH, U/I), magnesium (mmol/l), phosphorus (mmol/l), total bilirubin (μmol/l), total protein (g/l), triglycerides (TG, mmol/l) and uric acid (μmol/l). A comparative analysis was made between the indicators of the birds in their different habitats, which were conditionally divided into two areas. The Southeast region includes Sinite kamani NP (n = 23) and Stara Zagora (n = 1), Northwest includes Vrachanski Balkan NP (n = 8), Troyan (n = 1) and Sofia (n = 3). A comparison between the biochemical parameters of the birds was also made with regards to the months in which the blood samples were obtained. The winter-spring season includes the months from February to May, the summer-autumn - from June to September. Results of all the determinations were presented as Means ± Standard Error with the minimum and maximum values. Significant difference was accepted at the probability level of p < 0.05. The study parameters were further analysed using one-way analysis of variance (ANOVA), provided in SPSS Statistics (SPSS-Inc., 2019, Chicago, USA).

## Results

We determined the mean blood biochemistry values of wild common ravens in Bulgaria (Table 1). In addition, a comparison was made between the values of biochemical indicators with regards to the habitat of the birds (Table 2). For this purpose, the two populations were conditionally divided into a Southeast population from the Kotlenska Stara planina region and a Northwest population from Vrachanska Stara planina region. Similar statistics were also conducted regarding the season in which the blood samples were obtained (winter-spring season and summer-autumn season) (Table 3).

## Discussion

Conservation of endangered bird species can be refined by expanding the knowledge regarding morbidity and mortality of this group of animals (Hernandez and Margalida 2009). Clinical haematology and biochemical blood tests are an integral part of the diagnostics of captive birds (Campbell and Ellis 2007). Every year, sick and injured birds are found in the wild and are subsequently included in breeding and reintroduction programmes (Heredia 2005). In order to provide appropriate medical care for such endangered species, veterinarian physicians need haematology and biochemical reference ranges on which to base their diagnoses and subsequent therapy (Dujowich et al. 2005). Data on some of the blood biochemical parameters of species from the Crow family are scarce or even absent. The present study is the first in which blood samples were taken from wild common ravens for biochemical sampling. The resulting average, minimum and maximum values are compared with respect to the territory of sample collection (and putative residence of the individuals, respectively) and to the season. Some of the biochemical blood parameters studied may prove to be extremely important for the diagnosis and interpretation of behavioural problems in wild birds kept in captivity (Ferrer 1990).

From the investigated blood parameters, the following belong to the group of liver indicators - albumin, ALP, ALT, AST, LDH, total bilirubin and total protein. The liver transaminases AST and ALT change their plasma concentrations according to the functional state of the liver (Forbes 2008). An increase in **aspartate aminotransferase (AST)** is found in various tissue injuries, making it a non-specific indicator of liver injury (Harr 2006). Compared to values obtained for other corvids, the AST levels of our common ravens were high with average values of 459.89 ± 21.27 U/I and minimum/maximum from 171.00 to 674.00 U/I (Ihedioha et al. 2010). The results depended on the region and season of study. The **alanine aminotransferase (ALT)** levels are identical to those of other members of the Crow family. In birds, unlike mammals, significant increases in the total **alkaline phosphatase (ALP)** can be associated solely with increased osteoblastic activity (Mohanapriya et al. 2020). It is indicative as a marker of liver damage when seen in association with changes in the bile acids. The ranges obtained for ravens significantly exceeded those for magpies and they were affected by both range and season. They had an average value of 333.64 ± 60.46 U/I with fluctuations between 18.00 and1567.00 U/I. The normal serum levels of **lactate dehydrogenase (LDH)** in birds are between 70 and 400 U/l (Baldrey 2012). Compared to reference values for LDH in captive birds (Harr 2002), the common ravens in this study had higher levels. This parameter can be affected by capture and handling stress, which are often more profound in wild-caught compared to captive birds of the same species (Naidoo et al. 2008). The values of this biochemical indicator were also affected by the territory of blood collection. Elevated serum levels of LDH may also occur in liver disease, heart or muscle damage. The **bilirubin** in birds is of little diagnostic value in liver disease, unlike in mammals (Samour et al. 2015). The major bile pigment in birds is biliverdin, which is not metabolised to bilirubin (Samour et al. 2015). The normal range of serum **protein** is 3.5-5.5 mg%, with the percentage varying for different bird species (Sakas 2002). The obtained values were higher compared to other representatives of the Crow family. The measuring of serum **creatine kinase (CK)** is used in determining muscle and liver diseases in birds. Normal CK values are in the range 100-300 U/l (Baldrey 2012). Since creatine kinase is found primarily in cardiac and skeletal muscle, elevations are usually associated with skeletal muscle trauma (injections, feather plucking) and myocardial disease. The likely reason for the high values in the common ravens is their capture and the venipuncture itself. The CK values were also determined by the season and area of sampling. Elevated bile acids are considered a specific marker of liver dysfunction and **cholesterol** can be elevated or decreased as a result of liver disease (Harr 2006). Normal values of serum cholesterol in birds are not well studied, but vary between 100 and 300 mg%. This is also confirmed by the present study where its values are region and season dependent. Elevated levels may be seen in birds on high-fat diets, overweight birds and birds with hypothyroidism. Low levels can be seen in birds with liver and kidney disease. In cases of hepatic lipidosis, the cholesterol is often elevated along with **triglycerides (TG)** and liver markers (Samour et al. 2015). Blood concentrations of sodium, potassium, chloride, phosphorus and ionised calcium may be related to dysfunction of the kidneys, liver, adrenal glands and gastrointestinal tract and they may be influenced by diet and certain environmental factors. In similar studies in birds, none of the electrolyte concentrations was affected by age or sex. Only **chlorides** depend on the habitat of the birds (Heatley et al. 2013). These results are similar for the common ravens in this study, as their levels depended not only on the season, but also on the area of blood collection. In birds, as in mammals, one-third of the **calcium** is found bound to plasma proteins, so total calcium will be directly affected by plasma protein levels (Samour et al. 2015). Hypocalcemia together with an improper calcium to phosphorus ratio often indicates malnutrition (Baldrey 2012). In our study, calcium was not affected by habitat or season, unlike the **phosphorus** levels of the common ravens. The **magnesium** too was dependent on both territory and season of sampling. The average values of serum **amylase** in crows reach 1513.92 ± 72.23 U/l and the maximum - 2732 U/l. Our research results for the benchmark significantly exceed those reported by Baldrey (2012) - between 100 and 600 U/l. Elevated levels, up to three times the upper limit of the normal range, may be seen in acute pancreatitis. In some cases of enteritis, even in the absence of pancreatic lesions, amylase levels may be almost twice the upper limit, which could explain the results of this study. The common raven values depended on both the territory and the season of blood sampling. The **albumin** may increase due to dehydration in reproductively active females (Harr 2006). Birds with greater body mass maintain albumin levels in the upper limits. A decrease can be expected in the case of liver disease. In the Passeriformes order, part of which is the common raven, **glucose** values usually reach up to 16.65 mmol/l (Fokidis et al. 2011). The results obtained from our study were comparable. They were not affected by the region and season of sampling. Unlike mammals, where excess nitrogen is converted to urea, in birds, the main product of nitrogen catabolism is **uric acid** (Baldrey 2012). Uric acid is the major nitrogenous waste product in birds, which is why its serum levels are an indicator of kidney function. Normal values vary depending on the measurement technique, but are usually between 2 and 10 mg% (up to 15 mg% in some species). Uric acid levels in birds are affected by kidney function (Harr 2006), food intake (Samour et al. 2015) and the degree of hydration. Physiological hyperuricemia is observed in carnivore birds, so a fasting period before blood sampling is recommended in these species (Chitty 2018). Common ravens belong to this category of birds and their uric acid levels were relatively high. The maximum values reached up to 1625 μmol/l and fluctuations were relatively high. Unlike uric acid, **creatinine** levels in birds are of minimal diagnostic value. Plasma creatinine levels increase in the presence of a protein-rich diet and dehydration (Scope and Schwendenwein 2020).

## Conclusions

This is the first report on some basic biochemical blood parameters of healthy, wild common ravens in Bulgaria. The results provide an opportunity to expand further on the study, as well as to monitor and conserve the species populations. Most of the results obtained were similar to those published for other members of the Corvidae family, but there were also differences that may be explained by the capture- and handling stress, although others could be species-specific or the result of the different diet of the birds at a regional or national level. These results can be useful for the treatment and protection of the species in rescue and rehabilitation centres in the country and in Europe.

## Figures and Tables

**Table 1.** Average values of some blood biochemical parameters of common ravens in Bulgaria.

Serum biochemistry parameters	Mean ± Standard Error (n = 36)	Minimum and maximum values
AST U/I	459.89 ± 21.27	171.00-674.00
ALT U/I	85.75 ± 7.12	38.00-251.00
ALP U/I	333.64 ± 60.46	18.00-1567.00
LDH U/I	1035.78 ± 62.13	324.000-1757.00
CK U/I	897.81 ± 106.08	114.00-2511.00
Cholesterol mmol/l	5.02 ± 0.18	3.25-7.37
TG mmol/l	1.17 ± 0.05	0.67-2.02
Chloride mmol/l	115.11 ± 0.90	98.00-127.00
Amylase U/I	1513.92 ± 72.23	957.00-2732.00
Calcium mmol/l	2.17 ± 0.04	1.45-2.60
Phosphorus mmol/l	1.79 ± 0.30	0.39-6.97
Magnesium mmol/l	1.04 ± 0.04	0.66-1.45
Total protein g/l	44.30 ± 0.83	32.20-53.80
Albumin g/l	16.47 ± 0.35	8.60-21.60
Glucose mmol/l	16.41 ± 0.43	9.43-20.53
Total bilirubin μmol/l	12.23 ± 0.88	8.00-36.20
Creatinine μmol/l	39.58 ± 0.70	29.00-49.00
Uric acid μmol/l	672.85 ± 56.63	9.60-1625.00

**Table 2.** Average values of some blood biochemical parameters of common ravens in Bulgaria and statistically significant values (in bold) between the areas where the samples were taken.

Serum biochemistry parameters	Area	N	Mean	F	Sig.
AST U/I	Southeast	24	506.54	12.89	**0.00**
	Northwest	12	366.58		
ALT U/I	Southeast	24	94.63	3.31	0.08
	Northwest	12	68.00		
ALP U/I	Southeast	24	143.75	43.92	**0.00**
	Northwest	12	713.42		
LDH U/I	Southeast	24	926.54	7.29	**0.01**
	Northwest	12	1254.25		
CK U/I	Southeast	24	1083.92	7.26	**0.01**
	Northwest	12	525.58		
Cholesterol mmol/l	Southeast	24	4.64	11.53	**0.00**
	Northwest	12	5.79		
TG mmol/l	Southeast	24	1.14	0.72	0.40
	Northwest	12	1.23		
Chloride mmol/l	Southeast	24	117.17	14.30	**0.00**
	Northwest	12	111.00		
Amylase U/I	Southeast	24	1371.33	9.74	**0.00**
	Northwest	12	1799.08		
Calcium mmol/l	Southeast	24	2.14	0.62	0.44
	Northwest	12	2.22		
Phosphorus mmol/l	Southeast	24	0.79	60.71	**0.00**
	Northwest	12	3.81		
Magnesium mmol/l	Southeast	24	0.93	29.27	**0.00**
	Northwest	12	1.25		
Total protein g/l	Southeast	24	44.14	0.07	0.79
	Northwest	12	44.63		
Albumin g/l	Southeast	24	16.16	1.59	0.22
	Northwest	12	17.08		
Glucose mmol/l	Southeast	24	16.88	2.43	0.13
	Northwest	12	15.47		
Total bilirubin μmol/l	Southeast	24	12.35	0.04	0.85
	Northwest	12	11.99		
Creatinine μmol/l	Southeast	24	39.63	0.01	0.93
	Northwest	12	39.50		
Uric acid μmol/l	Southeast	24	671.29	0.00	0.97
	Northwest	12	675.97		

**Table 3.** Average values of some blood biochemical parameters of common ravens in Bulgaria and statistically significant values (in bold) with regards to the season of sample collection.

Serum biochemistry parameters	Season	N	Mean	F	Sig.
AST U/I	winter-spring	24	520.58	29.59	**0.00**
	summer-autumn	12	338.50		
ALT U/I	winter-spring	24	92.79	2.01	0.17
	summer-autumn	12	71.67		
ALP U/I	winter-spring	24	169.75	24.61	**0.00**
	summer-autumn	12	661.42		
LDH U/I	winter-spring	24	975.83	1.91	0.18
	summer-autumn	12	1155.67		
CK U/I	winter-spring	24	1159.17	18.06	**0.00**
	summer-autumn	12	375.08		
Cholesterol mmol/l	winter-spring	24	4.66	9.92	**0.00**
	summer-autumn	12	5.74		
TG mmol/l	winter-spring	24	1.11	2.41	0.13
	summer-autumn	12	1.28		
Chloride mmol/l	winter-spring	24	116.50	5.31	**0.03**
	summer-autumn	12	112.33		
Amylase U/I	winter-spring	24	1372.17	9.59	**0.00**
	summer-autumn	12	1797.42		
Calcium mmol/l	winter-spring	24	2.15	0.13	0.72
	summer-autumn	12	2.19		
Phosphorus mmol/l	winter-spring	24	0.81	54.01	**0.00**
	summer-autumn	12	3.76		
Magnesium mmol/l	winter-spring	24	0.93	26.08	**0.00**
	summer-autumn	12	1.24		
Total protein g/l	winter-spring	24	44.35	0.01	0.94
	summer-autumn	12	44.21		
Albumin g/l	winter-spring	24	16.35	0.23	0.63
	summer-autumn	12	16.71		
Glucose mmol/l	winter-spring	24	16.82	1.78	0.19
	summer-autumn	12	15.60		
Total bilirubin μmol/l	winter-spring	24	11.39	1.86	0.18
	summer-autumn	12	13.91		
Creatinine μmol/l	winter-spring	24	39.42	0.11	0.74
	summer-autumn	12	39.92		
Uric acid μmol/l	winter-spring	24	696.29	0.34	0.57
	summer-autumn	12	625.97		
